# Impact of continuous pharmaceutical care led by clinical pharmacists during transitions of care on medication adherence and clinical outcomes for patients with coronary heart disease: a prospective cohort study

**DOI:** 10.3389/fphar.2023.1249636

**Published:** 2023-08-23

**Authors:** Lingyan Gao, Yalei Han, Zhankun Jia, Pengfei Wang, Meijing Zhang, Teng Ma, Suying Yan, Hua Liu

**Affiliations:** ^1^ Department of Pharmacy, Aerospace Center Hospital, Beijing, China; ^2^ Department of Cardiology, Aerospace Center Hospital, Beijing, China; ^3^ Department of Pharmacy, Xuanwu Hospital Capital Medical University, Beijing, China

**Keywords:** coronary heart disease, transitions of care, continuous pharmaceutical care, cohort study, medication adherence, clinical pharmacist

## Abstract

**Objectives:** The study aimed to explore the impact of a continuous pharmaceutical care (CPC) program during care transitions on medication adherence and clinical outcomes for patients with coronary heart disease (CHD).

**Methods:** A prospective cohort study was conducted from April 2020 to February 2021. Patients diagnosed with CHD were selected and divided into intervention (CPC) and usual care (UC) groups by nurses at equal intervals based on admission time. The intervention group received CPC services provided by clinical pharmacists (including medication reconciliation, disease education, medication guidance, lifestyle counseling, and follow-up services) and usual care. The UC group received only routine medical care. The study compared medication adherence, clinical indicators (low-density lipoprotein cholesterol [LDL-C], blood pressure [BP], glycated hemoglobin [HbA1c] control rates), the incidence of adverse drug reactions (ADRs), and readmission rates (overall, major adverse cardiovascular events [MACEs]-related, and CHD risk factors-related) at admission and 1, 3, and 6 months after discharge between the two groups.

**Results:** A total of 228 patients with CHD completed the study, including 113 patients in the CPC group and 115 patients in the UC group. There were no significant differences (*p* > 0.05) in both groups in demographic and clinical characteristics at baseline. A total of 101 drug-related problems were identified in the CPC group (an average of 0.89 per person). The CPC group showed significantly higher medication adherence at 1, 3, and 6 months after discharge than the UC group (*p* < 0.05). At 3 and 6 months after discharge, the intervention group had significantly higher control rates of LDL-C (61.11% vs. 44.64% at 3 months, 78.18% vs. 51.43% at 6 months), and BP (91.15% vs. 77.39% at 3 months, 88.50% vs. 77.19% at 6 months). The CPC group had higher HbA1c control rates (53.85% vs. 34.21% at 3 months, 54.05% vs. 38.46% at 6 months) than the UC group. However, the differences were not statistically significant. The incidence of ADRs 6 months after discharge was significantly lower in the CPC group than in the UC group (5.13% vs. 12.17%, *p* < 0.05). The CPC group had a lower overall readmission rate (13.27% vs. 20.00%), MACE-related readmission rate (5.31% vs. 12.17%), and readmission rate related to CHD risk factors (0.88% vs. 2.61%) 6 months after discharge compared to the UC group. However, these differences were not statistically significant (*p* > 0.05).

**Conclusion:** CPC led by clinical pharmacists during care transitions effectively improved medication adherence, safety, and risk factor control in patients with CHD.

## Introduction

In recent years, the morbidity and mortality of coronary heart disease (CHD) have increased yearly, seriously affecting public health and raising medical expenses. International guidelines recommend that patients with CHD should take long-term secondary prevention drugs, such as antiplatelets, β-blockers (BB), angiotensin-converting enzyme inhibitors/angiotensin receptor blockers (ACEIs/ARBs), and statin lipid-lowering drugs (LLDs) and control risk factors, such as smoking (Expert Committee on Rational Drug Use of National Health and Family Planning Commission of the P. R. China, and the Chinese Pharmacists Association, 2018; Knuuti et al., 2020; Lawton et al., 2022). However, medication adherence in patients with CHD is generally low (Rahhal et al., 2021; Heitmann et al., 2022). A study showed that the medication non-adherence rate in patients of a large tertiary care health system after myocardial infarction (MI) was as high as 42.7% during 1-year follow-up (Crowley et al., 2015). A retrospective population-based cohort study was conducted to analyze medication adherence for secondary prevention after MI (Huber et al., 2019). Using a large Swiss medical claims database, they estimated the association between medication adherence and mortality and major adverse cardiovascular events (MACE). A high proportion of patients with low medication adherence was observed for all drug classes: 47.6% for dual-antiplatelet therapy (DAPT), 23.5% for LLDs, 47.3% for ACEI/ARB, and 88.1% for BBs. Patients with high adherence to DAPT, LLDs, and ACEI/ARB had a significantly reduced risk of all-cause mortality and MACE (LLD-group). Lack of knowledge about CHD and medications is the main factor that affects adherence (Zhao et al., 2015; Yu et al., 2022; Desai et al., 2023). In addition, the lack of education of physicians to patients on drug therapies and discharge follow-up are other important reasons for non-adherence (Ni et al., 2019).

Several studies have shown that clinical pharmacist interventions can significantly improve patient knowledge of CHD and secondary prevention drugs, leading to improved medication adherence (Ho et al., 2014; Ahmed Casper et al., 2022; Hong et al., 2022; Weeda et al., 2023). Pharmacist interventions can promptly detect and resolve adverse drug reactions (ADRs) and better control patient risk factors and clinical parameters (Phatak et al., 2016; Casper et al., 2019). A randomized controlled trial described a pharmaceutical care program in which pharmacists were trained to use motivational interviewing to follow up and educate patients with CHD after discharge (Ostbring et al., 2021). Compared to the standard care group, the pharmacist intervention group significantly improved patient adherence to LLDs (88% vs. 77%; *p* = 0.033) and aspirin (97% vs. 91%; *p* = 0.036). A retrospective cohort study was conducted to implement an outpatient Complex Coronary Interventions Medication Therapy Management (CCI-MTM) program in patients after percutaneous coronary intervention (PCI) in a Chinese hospital (Zhang et al., 2022). Compared to the usual care group, the proportion of patients who reached the LDL-C (73.8% vs. 41.0%, *p* < 0.001) and heart rate (14.8% vs. 4.1%, *p* = 0.007) goals in the PPCM was significantly higher. The median time to achieve the LDL-C goal was shorter in the PPCM group (31 days vs. 126 days, *p* = 0.001). The utilization rates of BBs (73.8% vs. 56.6%, *p* = 0.005) and ACEIs/ARBs (72.1% vs. 56.6%, *p* = 0.018) were higher in the PPCM group than in the UC group. There were no significant differences in ADRs between the two groups.

Transitions of care refer to a change in space (community to hospital, ward change, hospital to community) or staff while a patient is receiving care (World Health Organization, 2016). During this change process, poor medical information communication can easily lead to drug-related problems (DRPs) (Santell, 2006). It was estimated that approximately 46% of medication errors occur during care transitions (Pronovost et al., 2003). In 2019, the World Health Organization released “Medication Safety in Transitions of Care,” recommending that organizations implement a structured medication reconciliation process in care transitions (World Health Organization, 2019). Medication reconciliation includes patient interviews to collect an accurate medication history, coordinate and update medication lists, communicate medication changes with patients and caregivers, and ensure that patients have a current list of medications and are using their medications safely. The Chinese government also states that it is necessary to provide continuous and systematic services for prevention, treatment, rehabilitation, and health promotion, improve the fairness, accessibility, and effectiveness of health services, and achieve early diagnosis, early treatment, and early rehabilitation (China National Health and Family Planning Commission and the State Administration of Traditional Chinese Medicine, 2017; China National Health Commission, 2020).

Most CHD-related pharmaceutical care services focus only on CHD or a segment of care transitions (Scott et al., 2007; Phatak et al., 2016; Ostbring et al., 2021). Few studies have focused on patients with CHD with multiple chronic diseases and evaluated the outcomes of transition care interventions on risk factor control, MACE, and rehospitalization rate. We established a continuous pharmaceutical care (CPC) program for CHD patients. CPC is patient-centered and focuses on disease management and risk factor control at all stages of care transitions (Chen et al., 2022). The program aimed to improve medication adherence and secondary prevention risk factors, which could affect clinical outcomes such as re-hospitalization.

## Materials and methods

### Study design, participants, and setting

This prospective cohort study was conducted at the Aerospace Center Hospital in Beijing, China. The study subjects were patients admitted to the Department of Cardiology from April 2020 to February 2021. The inclusion criteria were patients who 1) were >/ = 18 years old, 2) had CHD (>50% stenosis in a major coronary vessel revealed by coronary angiography), and 3) had comorbidities of hypertension, diabetes, or dyslipidemia. Exclusion criteria were patients 1) with severe liver failure (prothrombin time activity <40%) or severe kidney dysfunction (creatinine clearance rate <30 mL/min), 2) with malignant tumors, 3) with communication disorders, and 4) refusal to give informed consent.

The eligible patients were divided into the CPC and the usual care (UC) groups by nurses at equal intervals based on admission time. UC patients only received routine medical care. This study was approved by the Hospital Medical Ethics Committee (20200331-QNCX-01, approval date 31 March 2020). Written informed consent was obtained from the patients.

### Components of continuous pharmaceutical care program for CHD patients

The CPC program consists of five steps to ensure comprehensive patient care. Step 1: Admission Medication Reconciliation (within 24 h): The pharmacist collaborates with the clinical team to conduct medical rounds from Monday to Friday to obtain information on the patient’s condition and the current drug treatment plan. Subsequently, the pharmacist consults with the patient face-to-face to gather a medication history. The pharmacist identifies DRPs and proposes interventions to physicians. The DRPs were categorized using the Pharmaceutical Care Network Europe DRP classification (version 9.0, Pharmaceutical Care Network Europe, 2020). Step 2: Patient Education (within 24 h of coronary angiography, lasting 30–90 min): The pharmacist provides a comprehensive education to the patient, including the disease state, such as the pathogenesis of CHD, risk factors (such as dyslipidemia, hypertension, diabetes, smoking), and monitoring indicators (such as LDL-C levels). The pharmacist also educates the patient about their medications, including drug names, purposes, usage and dosage instructions, duration of treatment, common ADRs, and precautions. Additionally, lifestyle guidance is tailored to the patient’s circumstances, covering diet, exercise, smoking cessation, alcohol consumption, and weight control. Step 3: Discharge Medication Reconciliation and Written Instructions (within 24 h before discharge, lasting approximately 30 min): The pharmacist organizes the patient’s discharge medications in a table format, including the generic name, brand name, specifications, the purpose of the drug, instructions on how to take the medication (before meals, with meals, after meals, before bedtime, *etc.*), and dosage. Key points specific to certain drugs are emphasized, such as monitoring the stool for melena when taking antiplatelet drugs, contacting the pharmacist or doctor for a persistent dry cough after taking an ACEI, and the need to monitor LDL-C levels, liver enzymes, and creatine kinase for statin use. Other key points include monitoring heart rate when taking β-blockers, blood pressure (BP) when taking antihypertensive drugs, and fasting and postprandial blood sugar levels when taking hypoglycemic drugs. Lifestyle guidance, such as weight control, moderate exercise, and a low-salt, low-fat diet, is also provided. Specific dietary recommendations are made for patients with diabetes or impaired glucose tolerance, advising them to consume fewer foods with a high glycemic index. Step 4: Verbal Education on Discharge Day (15–30 min): On the day of discharge, the pharmacist provides the patient with a guide sheet that outlines the medication regimen for the post-discharge period. The pharmacist explains the contents of the guide sheet in person, using actual medications as visual aids. The patient’s questions or concerns are addressed to ensure complete understanding. Contact information, such as phone numbers or WeChat accounts, is exchanged between the pharmacist and the patient for ongoing communication and support. Step 5: Follow-up Services at 1, 3, and 6 Months: These follow-ups may occur through “Physician-Pharmacist Joint Clinics”, “Pharmacy Clinics”, WeChat, or telephone communication. The purpose is to assess medication adherence, identify DRPs, review LDL-C levels, BP, and blood glucose, and make the necessary adjustments to the patient’s drug treatment plan. The pharmacist also emphasizes the importance of lifestyle improvements, including dietary modifications and exercise routines.

### Primary and secondary endpoints

The primary endpoint was medication adherence at admission and 1, 3, and 6 months after discharge. Medication adherence was assessed utilizing the visual analog scale (VAS), a self-reported measure of medication adherence (Amico et al., 2006). The VAS involved patients marking a line at a specific point on a continuum ranging from 0 to 100, indicating their adherence to their physician’s instructions over the past 4 weeks. A score of 0 meant that the patient did not follow the doctor’s instructions, while a score of 100 indicated strict adherence to the doctor’s instructions. Any score below 80 was classified as medication nonadherence (Liu et al., 2023). Secondary endpoints were the percentages of patients who reached the clinical goal parameters at admission and 1, 3, and 6 months after discharge. These included LDL-C, BP, and glycated hemoglobin (HbA1c). LDL-C and HbA1c were collected from laboratory tests. BPs were measured at each visit by physicians or nurses.

The goals were LDL-C<1.8 mmol/L or a decrease of >50% from baseline (Joint Committee Issued Chinese Guidelines for Prevention and Treatment of Dyslipidemia, 2016), BP < 140/90 mmHg or BP < 130/80 mmHg in diabetic patients (Expert Committee on Rational Drug Use of National Health and Family Planning Commission of the P. R. China, and the Chinese Pharmacists Association, 2018), and HbA1c<7.0% (Expert Committee on Rational Drug Use of National Health and Family Planning Commission of the P. R. China, and the Chinese Pharmacists Association, 2018). The incidence of ADRs and rehospitalization rate [overall rehospitalization, rehospitalization due to major adverse cardiac events (MACEs), rehospitalization due to CHD risk factors] within 6 months after discharge were also analyzed. The severity of ADRs was assessed according to the Common Terminology Criteria for Adverse Events version 5.0 (CTCAE v.5.0) (U.S. Department of Health and Human Services, 2017). MACEs included recurrent angina, fatal or non-fatal myocardial infarction, and revascularization (Xu and Li, 2019).

### Sample size calculation

Based on a previous study, the intervention group showed a high medication adherence rate of 53.85%, while the UC group had an adherence rate of 26.09% (Zhang et al., 2021). The minimum sample size required for each group was 77 (α = 0.05, 1:1 ratio between the intervention and control groups, β = 0.1, and a 20% loss-to-follow-up rate) using PASS (version 15).

### Statistical analysis

Microsoft Excel 2007 and SPSS 17.0 statistical software were used for data processing and analysis. Missing data was a problem in the collected data. The effect of the CPC program was evaluated by per-protocol analysis (PPA) and intention-to-treat analysis (ITT) (DeSouza et al., 2009). The last observation-carried-forward (LOCF) method was performed to replace missing post-baseline values for each visit (Bringsvor et al., 2018). The results of the ITT analysis are shown in the Supplementary Materials.

All continuous variables are expressed as mean ± standard deviation (‾x ± s). The normality of the data was assessed using the Kolmogorov-Smirnov test. When the quantitative data conformed to normal distributions, independent-sample t-tests were utilized. When the measurement data did not conform to normal distributions, the information was expressed as medians (interquartile ranges), and the data were analyzed using the Mann-Whitney *U* test. The categorical data are described as numbers and percentages (n, %), and the Pearson Chi-square test or the continuity-adjusted Chi-square test was estimated. A *p*-value <0.05 was considered statistically significant.

## Results

### Baseline demographic and clinical characteristics of patients

**FIGURE 1:**
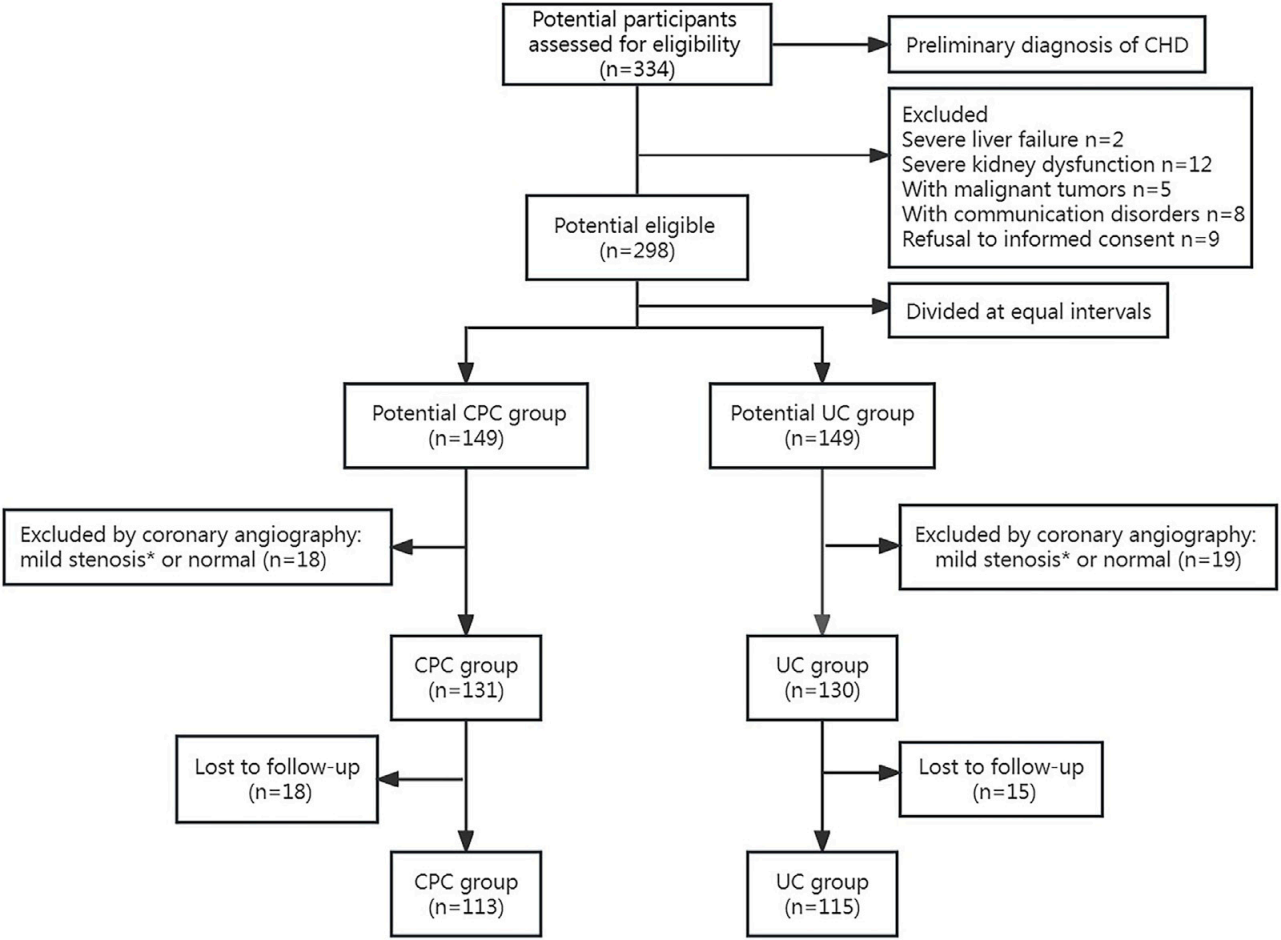
Flow chart of patient selection. * <50% stenosis in a major coronary vessel revealed by coronary angiography.

Figure 1 shows the flow of patients. Initially, 334 patients with a preliminary diagnosis of CHD were screened, and 36 were excluded based on the exclusion criteria. The remaining 298 eligible patients were divided into the CPC group (n = 149) and the UC group (n = 149). Subsequently, after undergoing coronary angiography, 131 patients from the CPC group (113 participated in the final follow-up) and 130 from the UC group (115 involved in the last follow-up) were included in the study. Demographics and other clinical characteristics of the patients at baseline are shown in Table 1. There were no significant differences in age, sex, comorbidities, smoking, drinking, medication adherence, blood lipids, BP, and blood glucose levels between the two groups (*p* > 0.05).

**TABLE 1:** Baseline demographic and clinical characteristics of patients.

Variable	CPC group (n = 113)	UC group (n = 115)	*p*-Value
Age (years) (mean ± SD)	60.80 ± 10.56	60.80 ± 9.59	0.804^a^
Male/female, n	76/37	71/44	0.384^b^
Current-smoker/non-smoker, n	41/72	42/73	0.970^b^
Drinking/non-drinking, n	36/77	35/80	0.816^b^
Dyslipidemia, n (%)	82 (72.57)	83 (72.17)	0.947^b^
Hypertension, n (%)	81 (71.68)	71 (61.74)	0.111^b^
Diabetes, n (%)	43 (38.05)	45 (39.13)	0.867^b^
Adherence (mean ± SD)	79.54 ± 10.69	79.22 ± 12.32	0.725^a^
Triglycerides (mmol/L)	2.09 ± 1.29	1.99 ± 1.26	0.383^a^
Total cholesterol (mmol/L)	4.72 ± 1.06	4.71 ± 1.19	0.978^c^
LDL-C (mmol/L)	2.68 ± 0.88	2.68 ± 0.89	0.955^c^
HDL-C (mmol/L)	1.03 ± 0.23	1.06 ± 0.24	0.364^a^
Systolic BP (mmHg)	134.66 ± 17.79	134.78 ± 21.02	0.763^a^
Diastolic BP(mmHg)	77.57 ± 10.93	76.78 ± 12.26	0.611^c^
FPG (mmol/L)	7.03 ± 2.22	7.48 ± 2.70	0.378^a^
HbA1c (%)	6.62 ± 1.47	6.76 ± 1.61	0.463^a^

SD: standard deviation, LDL-C: low-density lipoprotein cholesterol, BP: blood pressure, HbA1c: glycated hemoglobin, FPG: fasting plasma glucose.

^a^ Mann-Whitney *U* test.

^b^ Pearson Chi-square test.

^c^ Independent-sample *t*-test.

### Analysis of drug-related problems

In the CPC group, pharmacists identified 101 DRPs (0.89 per person). Among these, 57 (56.44%) were identified within 24 h after admission, 18 (17.82%) during hospitalization, 3 (2.97%) at discharge, and 23 (22.77%) in the community after discharge. Physicians accepted and successfully resolved 97 DRPs with an acceptance rate of 96.04%: 56 (98.25%, 56/57) during admission, 17 (94.44%, 17/18) during hospitalization, 3 (100.00%) at discharge, and 21 (91.30%, 21/23) in the community setting. The most common types of DRPs identified during admission were requiring additional drug therapies (43.86%, 25/57), drug omissions (21.05%, 12/57), inappropriate frequency (12.28%, 7/57), inappropriate dose (8.77%, 5/57), and inconsistent with the outpatient treatment plan (8.77%, 5/57). The DRPs during hospitalization consisted mainly of ADRs (44.44%, 8/18), requiring additional treatment regimens (27.78%, 5/18). In the community setting, most DRPs (69.57%, 16/23) were related to the need for additional drug therapies.

Examples of pharmacist’s interventions were 1) switching to higher intensity statins or combining ezetimibe therapy, 2) adding BBs, ACEIs/ARBs, or calcium-channel blockers, 3) increasing the dose of antihypertensive medications, 4) increasing the dose of hypoglycemic drugs or initiating new medications such as dapagliflozin.

### Primary endpoint-medication adherence

The CPC group consistently demonstrated significantly higher scores of medication adherence than the UC group (*p* < 0.001): 93.89 vs. 88.13 at 1 month, 95.58 vs. 89.27 at 3 months, and 97.18 vs. 89.94 at 6 months (Table 2).

**TABLE 2:** Comparison of medication adherence of patients between the two groups

Time	CPC group		UC group		*p*-Value
	mean ± SD	n	mean ± SD	n	
1 month	93.89 ± 7.21	113	88.13 ± 9.85	115	*<0.001^a^*
3 months	95.58 ± 6.25	113	89.27 ± 9.56	115	*<0.001^a^*
6 months	97.18 ± 5.03	113	89.94 ± 9.08	114^b^	*<0.001^a^*

SD: standard deviation.

^a^ Mann-Whitney *U* test; Number of missing data: b = 1.

The italic values mean statistically significant. A *p*-value <0.05 was considered statistically significant.

### Secondary endpoints in the two groups of patients

One month after discharge, there was no significant difference in the percentage of patients reaching the LDL-C goal between the CPC and UC groups (55.86% vs. 50.00%, *p* > 0.05). However, at 3 and 6 months after discharge, significantly more patients reached the LDL-C goal in the CPC group than in the UC group (61.11% vs. 44.64% at 3 months, *p* = 0.014; 78.18% vs. 51.43% at 6 months, *p* < 0.001). Details are shown in Table 3.

**TABLE 3:** Comparison of patients reaching the LDL-C goal between the two groups.

Time	CPC group		UC group		*χ* ^2^	*p*-Value
	n (%)	n	n (%)	n		
1 month	62 (55.86)	111^b^	54 (50.00)	108^e^	0.753	0.385^a^
3 months	66 (61.11)	108^c^	50 (44.64)	112^d^	5.982	*0.014^a^*
6 months	86 (78.18)	110^d^	54 (51.43)	105^f^	16.927	*<0.001^a^*

LDL-C: low-density lipoprotein cholesterol.

^a^ Pearson Chi-square test; Number of missing data: b = 2, c = 5, d = 3, e = 7, f = 10.

The italic values mean statistically significant. A *p*-value <0.05 was considered statistically significant.

One month after discharge, there was no significant difference in the percentage of patients reaching the BP goal between the CPC and UC groups (85.84% vs. 80.87%, *p* = 0.314). However, at 3 and 6 months after discharge, significantly more patients reached the BP target in the CPC group than in the UC group (91.15% vs. 77.39% at 3 months, *p* = 0.004; 88.50% vs. 77.19% at 6 months, *p* = 0.024). Details are shown in Table 4. At 3 and 6 months after discharge, more patients with diabetes reached the HbA1c goal in the CPC group than in the UC group (53.85% vs. 34.21% at 3 months, 54.05% vs. 38.46% at 6 months), but there was no statistical difference (*p* > 0.05). Details are shown in Table 5.

**TABLE 4:** Comparison of patients reaching the blood pressure goal between the two groups.

Time	CPC group		UC group		*χ* ^2^	*p*-Value
	n (%)	n	n (%)	n		
1 month	97 (85.84)	113	93 (80.87)	115	1.014	0.314^a^
3 months	103 (91.15)	113	89 (77.39)	115	8.115	*0.004^a^*
6 months	100 (88.50)	113	88 (77.19)	114^b^	5.095	*0.024^a^*

^a^ Pearson Chi-square test; Number of missing data: b = 1.

The italic values mean statistically significant. A *p*-value <0.05 was considered statistically significant.

**TABLE 5:** Comparison of patients with diabetes reaching the HbA1c goal between the two groups.

Time	CPC group		UC group		*χ* ^2^	*p*-Value
	n (%)	n	n (%)	n		
3 months	21 (53.85)	39^b^	13 (34.21)	38^d^	3.009	0.083^a^
6 months	20 (54.05)	37^c^	15 (38.46)	39^c^	1.858	0.173^a^

HbA1c: glycated hemoglobin.

^a^ Pearson Chi-square test; Number of missing data: b = 4, c = 6, d = 7.

### Comparison of adverse drug reactions between the two groups

Six months after discharge, the overall incidence of ADR in the CPC group was significantly lower than in the UC group [5.31% (6/113) vs. 13.04% (15/115), *p* = 0.043). The most frequent ADR in both groups was liver enzyme elevation. Details are shown in Table 6. In the CPC group, 4 ADRs (66.67%) were categorized as grade 1, and 2 ADRs (33.33%) were classified as grade 2. While in the UC group, 6 ADRs (40.00%) were categorized as grade 1, 8 ADRs (53.33%) were classified as grade 2, and 1 ADR (6.67%) was categorized as grade 3.

**TABLE 6:** Comparison of adverse drug reactions between the two groups at 6-month follow-up.

Group	Adverse drug reactions, n (%)								
	Liver enzyme elevation	Rash	Dry cough	Local bleeding	Headache	Abdominal distention	Gastrointestinal bleeding	Insomnia	Total
CPC group	2 (1.77)	1 (0.88)	1 (0.88)	1 (0.88)	0 (0)	1 (0.88)	0 (0)	0 (0)	6 (5.31)
UC group	8 (6.96)	0 (0)	0 (0)	0 (0)	3 (2.61)	2 (1.74)	1 (0.87)	1 (0.87)	15 (13.04)
*χ* ^2^	—	—	—	—	—	—	—	—	4.077
*p*-value	—	—	—	—	—	—	—	—	*0.043^a^*

^a^ Pearson Chi-square test.

The italic values mean statistically significant. A *p*-value <0.05 was considered statistically significant.

### Comparison of rehospitalization of patients in the two groups

Six months after discharge, the rates of overall rehospitalization (13.27% vs. 20.00%), rehospitalization due to MACE (5.31% vs. 12.17%), and rehospitalization due to risk factors for CHD (0.88% vs. 2.61%) were lower in the CPC group than in the UC group. However, the differences were not statistically significant (*p* > 0.05). Details are shown in Table 7.

**TABLE 7:** Comparison of rehospitalizations between the two groups at 6-month follow-up.

	CPC group (n = 113)	UC group (n = 115)	*p*-Value
MACEs, n (%)	6 (5.31)	14 (12.17)	0.067^a^
Risk factor, n (%)	1 (0.88)	3 (2.61)	0.626^b^
Others, n (%)	8 (7.08)	6 (5.22)	0.558^a^
Total, n (%)	15 (13.27)	23 (20.00)	0.173^a^

MACE: major adverse cardiac event.

^a^ Pearson Chi-square test.

^b^ Continuity-adjusted Chi-square test.

## Discussion

This study focused on implementing a continuous pharmaceutical care program during care transitions, which proved beneficial in several aspects. This proactive approach to managing medication therapy positively impacted medication adherence and facilitated better disease management and control of risk factors associated with the patient’s condition.

### The impact of continuous pharmaceutical care program on identifying drug-related problems

Analysis of DRP distribution during the care transition revealed that 56.44% of DRPs occurred within 24 h after admission, 17.82% during hospitalization, 2.97% at discharge, and 22.77% in the community. This highlights the critical stages in which DRPs commonly arise during the transition. The hospital’s computerized physician order entry systems reduce medication errors during discharge. During admission, we observed that 21.05% of medication orders were missed due to poor communication between physicians and patients.

Additionally, physicians sometimes overlooked other chronic diseases such as hypertension, diabetes, dyslipidemia, and hyperuricemia when managing CHD patients in the community setting, resulting in a higher proportion (43.86%) of patients requiring additional treatments during admission. Effective communication between healthcare providers and patients about chronic diseases becomes crucial to address these challenges. DRPs during hospitalization focused primarily on ADRs (44.44%) and the need for additional treatment options (27.78%). Pharmacists played an essential role during ward rounds by identifying ADRs and providing feedback to physicians. This timely intervention allowed necessary adjustments to drug treatment plans, preventing patients from experiencing ADRs upon discharge. Community-based DRPs focused mainly on adding additional treatment plans (69.57%). After patients receive treatment plan adjustments during hospitalization, monitoring and managing their blood lipids, BP, blood glucose, and other laboratory indicators in the community setting becomes necessary. Subsequently, the treatment plans can be adjusted based on the parameter control.

### The impact of continuous pharmaceutical care program on improving patient medication adherence

Medication adherence is a cornerstone in assessing patient-centered care. Studies have confirmed that pharmacists can effectively enhance patients’ understanding of diseases and medication treatment plans through disease education and medication counseling, thus improving medication adherence (Phatak et al., 2016; Lee et al., 2019; Hong et al., 2022). The CPC program provides patients with multiple time points and frequencies of follow-up services, which led to significantly enhanced medication adherence at 1, 3, and 6 months follow-ups after discharge.

The CPC program allows pharmacists to establish therapeutic relationships with patients to enhance the patient’s healthcare experience and increase trust in pharmacists. Patients have gradually embraced the concept of pharmacist-led outpatient care and actively chosen pharmacy clinics for follow-up and medication advice, thus improving overall awareness of pharmacy clinics. Many patients seek pharmacist opinions through pharmacy clinics, WeChat, and telephone consultations, after physicians have adjusted their medication treatment plans. In this study, 56.41% of the patients in the CPC group chose the “physician-pharmacist joint clinic” for follow-up, 28.21% received follow-up through the “pharmacy clinic”, and 15.38% received follow-up via WeChat or telephone. This ensures medication safety and enhances pharmacists’ professional sense of value and identity.

### The impact of continuous pharmaceutical care program on patient clinical outcomes

Dyslipidemia, hypertension, and diabetes are recognized as risk factors for CHD (Expert Committee on Rational Drug Use of National Health and Family Planning Commission of the P. R. China, and the Chinese Pharmacists Association, 2018). Existing research consistently emphasizes the importance of intervention and control of these risk factors for secondary prevention of CHD (Huber et al., 2019; Sotorra-Figuerola et al., 2021; Zhang et al., 2021).

In this study, more patients in the CPC group achieved LDL-C and BP targets at 3 and 6-month follow-ups, leading to better control of these risk factors associated with CHD. CPC also significantly reduced the occurrence of ADRs at 6-month follow-up after hospital discharge. Although not statistically significant, the rates of overall readmissions, readmissions due to MACEs, and readmissions related to CHD risk factors in the CPC group were lower than in the UC group at the 6-month follow-up. These results show that the CPC program during the transitional period of medical care can play a role in improving the short-term prognosis of patients. These results are consistent with previous studies where pharmaceutical care services have been shown to positively impact reducing the incidence of ADRs and improving patients’ quality of life (Phatak et al., 2016; Sotorra-Figuerola et al., 2021).

The loss of patient follow-up can be attributed to two main reasons. First, in China, the role and benefits of pharmacists may be perceived by patients as insignificant. Instead, patients often prefer to consult a doctor for follow-up appointments directly. Second, attending the “physician-pharmacist joint clinic” or “pharmacy clinic” in our hospital for follow-ups is inconvenient for many patients, especially those from other provinces or cities. The issue of patients withdrawing from clinical follow-up is not unique to our hospital but is a more widespread concern. To address this, enhancing patient engagement and retention can be achieved through various approaches. One potential solution is to increase patient awareness of pharmacists’ vital role in their healthcare and the benefits of their participation in the follow-up process. Furthermore, improving the convenience of pharmacist visits, such as offering telemedicine options or establishing partnerships with pharmacies in patients’ hometowns, could encourage better patient participation and retention in follow-up care.

Data bias in this study was assessed through the following analyses. First, it should be noted that the research design in this study is non-randomized, which may introduce selection bias. However, to mitigate this, a nurse without involvement in the study was responsible for dividing eligible patients into the CPC and UC groups. The demographic and clinical characteristics of the two groups at baseline were relatively similar. Second, when evaluating medication adherence, a self-reported tool was utilized. Although this method is commonly employed, it does have the potential to introduce bias due to differences in subjective feelings and reporting tendencies among patients. Last, it is essential to address missing data, as analyses that overlook these gaps can lead to biased parameter estimates (Blazek et al., 2021). This study implemented the LOCF method to replace missing data and reduce bias (Bringsvor et al., 2018).

In this study, the economic costs and benefits associated with CPC are complex, with several considerations. First, the participation of pharmacists in the CPC group demonstrated a reduction in the incidence of adverse drug events, leading to potential cost savings by minimizing the subsequent expenses associated with managing these events. Second, uncontrolled dyslipidemia, hypertension, and diabetes are well-known risk factors for recurrent CHD. In cases where doctors overlooked or poorly controlled these risk factors, clinical pharmacists were able to identify meaningful interventions, which could result in increased drug costs for patients. However, it is essential to note that the CPC led by clinical pharmacists during care transitions improved medication adherence and helped patients achieve their treatment goals for modifiable risk factors such as LDL, BP, and HbA1c, all associated with CHD. Therefore, the higher cost of drugs in this context could be considered an investment to achieve effective secondary prevention of CHD. Third, it should be acknowledged that implementing the CPC program may significantly increase the medical workload. However, the crude cost calculations in this study did not incorporate the pharmacist’s time spent executing the CPC program, which involved hospital intervention and follow-up after discharge. As a result, accurately estimating the cost of this aspect remains a significant challenge and requires further methodology development. Given the complexity of the economic implications related to CPC, more comprehensive studies are needed in this field to understand the costs and benefits better.

This study has several limitations. It had a limited sample size and was conducted in a single center. Therefore, large-scale and multi-center studies are necessary to further validate the impact of the continuous pharmaceutical care program on care transitions. The study had a follow-up period of only 6 months, which may not be adequate to fully assess the long-term outcomes of the interventions.

## Conclusion

The continuous pharmaceutical care program provided for patients with CHD, from admission to hospitalization, discharge, and community care, resulted in improved medication adherence, enhanced control of risk factors such as lipids and blood pressure, and reduced the incidence of ADRs.

## Data Availability

The original contributions presented in the study are included in the article/Supplementary Material, further inquiries can be directed to the corresponding author.
