# Supplementary material for: Impact of continuous pharmaceutical care led by clinical pharmacists during transitions of care on medication adherence and clinical outcomes for patients with coronary heart disease: a prospective cohort study
Source: Front Pharmacol. 2023 Aug 23;14:1249636. doi: 10.3389/fphar.2023.1249636 (PMC10484794; doi:10.3389/fphar.2023.1249636)
Supplement: Supplementary file 1 [file Table1.DOCX]

**Table S1**. Comparison of medication adherence of patients between the two groups

| Time | CPC Group | |  | UC Group | | *P* value |
| --- | --- | --- | --- | --- | --- | --- |
|  | mean±SD | n |  | mean±SD | n |  |
| 1 month | 93.89±7.21 | 113 |  | 88.13±9.85 | 115 | *<0.001^a^* |
| 3 months | 95.58±6.25 | 113 |  | 89.27±9.56 | 115 | *<0.001^a^* |
| 6 months | 97.18±5.03 | 113 |  | 89.94±9.08 | 115 | *<0.001^a^* |

SD: standard deviation

^a^ Mann-Whitney U test

**Table S2**. Comparison of patients reaching the LDL-C goal between the two groups

| Time | CPC Group | |  | UC Group | | *χ*^2^ | *P* value |
| --- | --- | --- | --- | --- | --- | --- | --- |
|  | n (%) | n |  | n (%) | n |  |  |
| 1 month | 62(54.87) | 113 |  | 58(50.43) | 115 | 0.449 | 0.503^a^ |
| 3 months | 68 (60.18) | 113 |  | 52 (45.22) | 115 | 5.116 | *0.024^a^* |
| 6 months | 89 (78.76) | 113 |  | 61 (53.04) | 115 | 16.749 | *<0.001^a^* |

LDL-C: low-density lipoprotein cholesterol

^a^ Pearson Chi-square test

**Table S3**. Comparison of patients reaching the blood pressure goal between the two groups

| Time | CPC Group | |  | UC Group | | *χ*^2^ | *P* value |
| --- | --- | --- | --- | --- | --- | --- | --- |
|  | n (%) | n |  | n (%) | n |  |  |
| 1 month | 97 (85.84) | 113 |  | 93 (80.87) | 115 | 1.014 | 0.314^a^ |
| 3 months | 103 (91.15) | 113 |  | 89 (77.39) | 115 | 8.115 | *0.004^a^* |
| 6 months | 100 (88.50) | 113 |  | 89(77.39) | 115 | 4.956 | *0.026^a^* |

^a^ Pearson Chi-square test

**Table S4**. Comparison of patients with diabetes reaching the HbA1c goal between the two groups

| Time | CPC Group | |  | UC Group | | *χ*^2^ | *P* value |
| --- | --- | --- | --- | --- | --- | --- | --- |
|  | n (%) | n |  | n (%) | n |  |  |
| 3 months | 22 (51.16) | 43 |  | 18 (40.00) | 45 | 1.105 | 0.293^a^ |
| 6 months | 23 (53.49) | 43 |  | 16 (35.56) | 45 | 2.865 | 0.090^a^ |

HbA1c: glycated hemoglobin

^a^ Pearson Chi-square test
